# Promoting oral and dental health in early childhood - knowledge, views and current practices among paediatricians in Israel

**DOI:** 10.3389/fped.2022.956365

**Published:** 2023-01-06

**Authors:** Aviv Shmueli, Aida Assad-Halloun, Avia Fux-Noy, Elinor Halperson, Einat Shmueli, Diana Ram, Moti Moskovitz

**Affiliations:** ^1^ Faculty of Dental Medicine, Hebrew University and Hadassah, Jerusalem, Israel; ^2^ Paediatric Pulmonology Institute, Schneider Children's Medical Center of Israel, Petach tikva, Israel; ^3^ Sackler Faculty of Medicine, Tel Aviv University, Tel Aviv, Israel

**Keywords:** early childhood caries, paediatricians, caries risk assessment, dental education, dental health

## Abstract

Early childhood dental caries is the most prevalent disease in childhood. Paediatricians are considered by parents as the main authority on prevention and treatment of all the health requirements of their infants.

**Aim:**

The aim of this study was to evaluate the knowledge and practice of paediatricians in promoting oral and dental health among young patients (under age 36 months) and their parents.

**Materials and methods:**

145 anonymous questionnaires were distributed among paediatricians, 130 of them were 90% or above answered and were suitable for analysis for this study.75% of the questionnaires were distributed during the national convention of the Israeli Association of Clinical Paediatrics, 10% in paediatric ambulatory clinics and 15% in paediatric departments in hospitals. Questionnaires were distributed between 2018 and 2020. The inclusion criteria were physicians specialists in paediatrics or residents in paediatrics, all have Israeli licences to practice in Israel. exclusion criteria were partially filled questionnaires. The questionnaire was validated in a pilot study during the years 2010–2012. In addition to demographic variables that included medical training, post-graduate education and clinical practice the questionnaire included 42 questions. Eleven questions on demographics and amount of dental training during academic and clinical training 31 questions belonged to several sections that referred to the participants’ awareness of the AAP guidelines regarding oral and dental health and knowledge of oral health. In each section paediatricians were asked to answer or give an opinion on a specific issue, their answers were coded to scores on a scale of 0–5 and summed per section. Correlations between different variables were analysed. The *t*-test and Mann-Whitney *U* test were performed for comparing two variables. For comparing more than two variables, we used the Kruskal-Wallis one-way analysis of variance test or ANOVA.

**Results:**

The response rate was 89% (130 questionnaires out of 145). The survey showed that most paediatricians (80%) recognized their role in maintaining the oral and dental health of their young patients. Nevertheless, most admitted that they do not perform simple procedures on a regular basis, like dental examinations (64.6%), or asking parents about feeding habits (59.2%) or teeth brushing (75.4%). Only 21% of the participants expressed adequate knowledge of dental care for children younger than age 3 years. Fifty-eight percent of the participants never had any dental training during their entire paediatric medicine training, including medical school. Paediatricians in private or baby clinics received higher scores in practicing caries prevention, 24.15 ± 5.17 (SD), than paediatricians in hospitals, 2.79 ± 0.54 (SD) (*p* = 0.006). Caries prevention practice was not found to correlate with paediatricians’ knowledge or attitudes regarding oral and dental caries prevention.

**Conclusion:**

Oral and dental knowledge should be incorporated into the paediatric medicine curriculum. With their heavy workload, paediatricians generally do not implement dental caries risk assessment and counselling.

## Introduction

Early childhood dental caries (ECC) is an infectious multifactorial disease, and is the most common chronic disease in childhood, according to the U.S. Centre for Disease Control and Prevention (CDC) (1–3). Though the prevalence of dental caries in the general population has declined, this trend is not seen in early childhood caries (4, 5). In developed countries, the prevalence of early childhood caries has been reported as 1%–12%, but can reach 70% in high-risk populations, such as low socioeconomic groups or ethnic minorities (6).

Data regarding early childhood caries in Israel are limited. A survey conducted in nursery clinics in Jerusalem reported a prevalence of ECC of 15.3% (7). In a study of the Bedouin population in the periphery of Jerusalem, the prevalence of ECC was 17.6% (8). Of 1,210 6-year-old-children residing in 23 local authorities, 61.7% had dental tooth decay and only 38.3% were caries free (9). Although ECC is defined as dental caries before age 6 years, trends can be learned from data regarding the dental caries experience of children in their early years of life.

ECC is a virulent form of dental caries, which is biofilm (plaque)-induced acid demineralization of enamel or dentin, mediated by saliva. The disease is characterized by rapid and wide damage to the primary dentition soon after teeth eruption. ECC presents initially on the maxillary incisors and then spreads to the molars. Etiologic factors include inadequate feeding habits like nursing *ad libitum* and failure to maintain oral hygiene. In the past, ECC was termed baby bottle tooth decay. Today, ECC is defined as the presence of tooth decay (with or without cavitation) before age 6 years. Evidence of caries in smooth surfaces before age 3 years or a decay-missing-filled teeth score greater than child age in years is considered severe ECC.

The current treatment approach for ECC focuses on primary prevention (5). The American Academy of Paediatric Dentistry (AAPD) recommends assessing caries risk from age 6 months and providing instructions for caries prevention to infants’ parents. According to AAPD recommendations, children by their first birthday should be examined by a dentist, to establish dental home (1).

During early childhood, infants and their parents meet paediatricians routinely to follow growth and development. Parents consider the paediatrician as proficient in topics of prevention, early detection of medical problems and consultation for adequate infant care. While the paediatric dentist usually meets the infant after caries damage is already detectable, the paediatrician is in a key position for early examination, detection, and prevention (2, 5, 10).

In May 2003, the American Academy of Paediatrics (AAP) published a policy statement regarding oral and dental health of children (5). According to AAP recommendations, paediatricians and medical staff should incorporate caries risk assessment and caries preventive instructions routinely, even before infants reach age 6 months (5, 10). Several publications have supported implementation of the AAP recommendations for caries risk assessment. Dela Cruz (2004) (11) and Lewis CW et al.’ (12) found that most American paediatricians agree that early detection and prevention of dental problems is part of routine paediatric examination. Only 15% considered it necessary to refer infants to paediatric dentists by age 1 year (13).

In Israel, the degree to which paediatricians perform caries risk assessment and caries preventive consultation was not previously studied. The aim of this study was to evaluate the knowledge and practice of Israeli paediatricians regarding oral and dental health promotion among young patients (under age 36 months), and to evaluate barriers to such practice.

## Materials and methods

145 questionnaires were distributed between March 2018 and March 2020 among paediatricians at the national conventions of the Israeli Association of Clinical Paediatrics, in paediatric ambulatory clinics and in paediatric departments in hospitals. 75% of the questionnaires (98) were collected in the national convention of the Israeli Association of clinical Paediatrics 2018, 15% (19) were collected in Paediatric departments in hospitals and 10% (13) were collected in Paediatric ambulatory clinics. The questionnaires collected in Paediatric ambulatory clinics were given only to paediatricians who had attended the convention of the Israeli Association of Clinical Paediatrics but did not fill the questionnaire during the convention. The same researcher that distributed questionaries at the conventions collected the questionnaires at the other locations and answered technical questions the responders that came up. The questionnaire was anonymous, no name or Identification number was mentioned, every questionnaire was numbered with serial number for the analysis.

### Ethical considerations

The study protocol was approved by the Institutional Human Subjects Ethics Committee of Hadassah Medical Organization, Jerusalem, Israel (0429-16-HMO). The ethics committee exempt from signing consent form, since answering the questionnaire was voluntary the consent was received by the will of the pediatrician to fill the questionnaire.

### Validation

The questionnaire was based on similar studies (13, 14) that were conducted in the U.S. and on the AAP position paper regarding pediatrician’s role in caries risk assessment in children under 3 years (5). The questionnaires were distributed in a congress of the Israeli Association of Clinical Pediatrics among 87 pediatricians between 2010 and 2012 and the results were in accordance with previous studies (13, 14) (data not published). Based on that pilot study, we conducted the current study with the same questionnaire.

An introduction was added to the questionnaire explaining the identity of the researchers and the aims of the study. In addition, the researcher was present in the convention and was able to answer participants’ questions.

The questionnaire was composed of multiple-choice questions with four response options, and open questions, and was administered anonymously. It included 42 questions and statements: Eleven questions on demographics: age, gender, institute of residency and seniority in paediatrics, amount of dental training during academic and clinical training.

Variables regarding clinical practice: geographic region of working, type of clinic (private, national health service, nursery clinic, paediatric department in a hospital), the number of patients per day and the proportion of them under age 3 years.

The second part included 31 questions that referred to the participants’ awareness of the AAP guidelines regarding oral and dental health and Knowledge of oral health. It consisted of 5 statements, and the participants were asked to decide if they agree or disagree. To differentiate between the overall score between the variables, the answers of the participants were summarized into scores between 0 and 5, this score was referred as final score in knowledge of oral health. For example, if all the answers were right the final score in knowledge of oral health was 5. If all answers were wrong the score was 0.

The third part referred to attitudes regarding the role of paediatricians in promoting oral and dental health. It consisted of 5 statements and the participants were asked to grade their consent as “I do not agree”, “I partially agree” or “I agree.” “To correlate between variables, the answers of the participants were transformed into score between 1 and 3, this score 5–15) and were summarized as measured attitudes regarding the role of paediatricians in promoting oral and dental health”.

### The practice and promotion of oral health among children, and the obstacles encountered

Participants were asked about their confidence in providing oral health consultation and diagnosis, and about their knowledge regarding the Israeli Ministry of Health recommendations regarding oral health promotion among paediatric patients. Finally, the participants were asked if they would like to take part in a course about preventive dentistry for infants and toddlers.

There were 10 questions, for every question there were four options to answer: “Never”, “Hardly”, “Usually”, “Always” and “No opinion”. “Always” gave score of 4 and “never” gave score of 1. “No opinion” was scored zero. Statement scores were summed to give a possible score range of 10–40.

This score was referred as final score in practice and promotion of oral health among children.

### Statistics

Statistical analysis was performed only on questionnaires that were at least 90% filled out. From the 145 questionnaires that were distributed 130 (89.7%) were found suitable for statistical analysis. The other fifteen were excluded.

Data were analysed using SPSS 22.0 software (IBM Inc., Chicago, Ill., USA). The *t*-test and Mann-Whitney *U* test were performed for comparing two variables. For comparing more than two variables, we used the Kruskal-Wallis one-way analysis of variance test or ANOVA. In cases of statistical significance in the ANOVA test, correction to the degree of significance was performed by Scheffe or T3 Dunnett tests, to identify the contribution of each of the two variables to the correlation.

Correlations between two categorical variables were performed by *χ*^2^ and Fisher's exact test.

The level of statistical significance was set at *p* < 0.05.

## Results

The study population comprised paediatricians who were members of the Israeli Society of Clinical Paediatrics, and paediatricians who worked in one of several hospitals in Israel.

### Demographics

Of the 130 paediatricians included in the study, 53 (40.8%) were males and 77 (59.2%) females. The median age was 42 years (27–73 years). The range of professional experience was 0.5–50 years, the mean was 18.13 years, SD = 14.21. Only 82 paediatricians stated the country where they performed their residency: 48 (59%) graduated in Israel and 34 (41%) in other countries.

### Clinical practice

Paediatricians from the entire country participated in the study: 35% from the north, 23% from the centre, 15% from Jerusalem, and 10% from the south; 17% did not provide this information. Sixty-four reported working in hospitals, 43 in baby clinics, 27 in the national health service, 24 in independent clinics, and 6 in private clinics. Some of the paediatricians worked in more than one position.

### Patient populations

Paediatricians reported seeing 0–75 children in a day, 24 children on average; of whom 65% (SD = 25.25) on average were below the age 3 years (the value 0 is because some of the responders were retired from clinical work).

### Oral and dental health training during academic and clinical training

Data regarding dental training during the stages of clinical training are shown in Table 1.

**Table 1:** Reported oral health medical education during each professional training stage: the number of courses in medical school, residency and continuing education.

	No answer	More than one course	One course	No training
Medical school	2.3 (3)%	3.1 (4)%	20.8 (27)%	73.8 (96)%
Residency	6.2 (8)%	5.4 (7)%	10.0 (13)%	78.5 (102)%
Continuing education	4.6 (6)%	7.7 (10)%	13.1 (17)%	74.6 (97)%
Any training	2 (3)%	40 (52)%	58 (75)%

Most paediatricians (58%) did not receive any oral and dental health training during their clinical paediatric training. No correlation was found between dental training and professional seniority, stratified by 5 years and above, and below 5 years. The rationale for this analysis was the assumption that paediatricians with 5 years of experience or more are further from their basic paediatric training and may have been more exposed to continuing education.

### Oral and dental health knowledge

This was evaluated by participants stating whether they thought that each of five statements regarding oral and dental health was correct or incorrect. Only 27.7% (*n* = 36) reported familiarity with the AAP guidelines regarding oral and dental health; 66.9% (*n* = 87) were not familiar with the guidelines.

A significant positive correlation was found between dental education during the paediatrician’s clinical training and familiarity with AAP guidelines (OR 3.67; 95% CI: 1.59–8.33; *p* = 0.003).

No correlation was found between seniority in paediatrics, and oral and dental knowledge.

The results are shown in Table 2.

**Table 2:** Responders’ answers distribution to statements on oral and dental health knowledge.

Statement	Correct/Incorrect	Right answer	Wrong answer	No answer
Only bottle-fed infants develop early childhood caries	Incorrect	113 (86.9%)	17 (13.1%)	-
Fluoride supplementation is acceptable in geographic areas with partial fluoridation	Incorrect	54 (41.5%)	74 (56.9%)	2 (1.6%)
Cariogenic bacteria may transfer from mother to child	Correct	93 (71.5%)	32 (24.7%)	5 (3.8%)
The AAP recommends a paediatric dental examination at age 1 year	Correct	93 (71.5%)	32 (24.7%)	5 (3.8%)
Preterm babies are at higher risk for dental caries	Correct	83 (63.9%)	42 (32.3%)	5 (3.8%)

For each statement, the correct answer is given and the percentage of paediatricians that gave a right answer a wrong answer or no answer is given.

### Attitudes of paediatricians toward their role in promoting oral and dental health

This was evaluated by participants’ responses, in agreement or disagreement, with five declarations of the role of paediatricians in promoting oral and dental health among children younger than age 3 years (Table 3). A significant correlation (*p* = 0.027) was found between working in an independent office and agreement with the statement that paediatricians have a central role in promoting their patients’ oral and dental health.

**Table 3:** Responders’ answers distribution to the role of paediatricians in promoting oral and dental health.

Statement	Does not agree	Partially agrees	Agrees	No answer
Inspection of teeth is an integral part of a physical examination	9 (6.9%)	6 (4.6%)	113 (86.9%)	2 (1.5%)
Children from age 6 months should be routinely assessed for risks of early childhood caries	15 (11.5%)	36 (27.7%)	76 (58.5%)	3 (2.3%)
Guidance for caries prevention should be given during routine follow-up	3 (2.3%)	10 (7.7%)	115 (88.5%)	2 (1.5%)
Paediatricians have a central role in promoting their patients’ dental health	5 (3.8%)	20 (15.4%)	104 (80.0%)	1 (0.8%)
Every child should be referred to a paediatric dentist for examination at age 1 year	23 (17.7%)	40 (30.8%)	66 (50.8%)	1 (0.8%)

For each statement, the correct answer is given and the percentage of paediatricians that agreed, partially agreed, disagreed, or gave no answer is given.

### Clinical practice

Eighty-five percent (*n* = 111) of the participants in the survey reported that they provided the parents of their patients verbal consulting regarding oral and dental health and caries prevention. One participant reported providing parents with a demonstration and three reported giving a leaflet with information regarding caries prevention. Table 4 summarize responders’ answers distribution regarding clinical practice in dental caries prevention and treatment. We found a correlation between the number of patients younger than 36 months treated by paediatricians and favourable clinical practice regarding caries prevention (*p* = 0.005). Accordingly, paediatricians who examined more young children performed more caries prevention in their daily practice. We also found a positive correlation between paediatrician seniority and the tendency to practice caries prevention as part of routine practice (95% CI: 0.285–0.585; *p* ≤ 0.001). Paediatricians who work in private or baby clinics received higher scores in practicing caries prevention 24.15 ± 5.17 (SD), than paediatricians in hospitals, 2.79 ± 0.54 (SD) (*p* = 0.006). Correlations were not found of caries prevention practice with knowledge was (*p* = 0.148, rs = 0.128 by Pearson correlation coefficient) or with attitudes of the paediatrician about oral and dental caries prevention (*p* = 0.836 rs = 0.018 by Pearson correlation coefficient). A significant correlation was not found between consent with the statement that visual examination of teeth is part of the physical examination, and the actual performance of such examination (*p* = 0.46 by Fisher’s test). A significant correlation was found between consent with AAP recommendations and the referral of every child at age 12 months to a dental check-up (OR 10.8; 95% CI: 1.49–10; *p* = 0.001).

**Table 4:** Responders’ answers distribution regarding clinical practice in dental caries prevention and treatment.

Question	Never	Hardly	Usually	Always	No opinion
Do you inquire if an infant goes to sleep with a sweetened beverage?	15.4%	36.9%	31.5%	16.2%	
Do you instruct parents to avoid giving their children sweetened foods and beverages?	4.6%	29.2%	32.3%	35.6%	
Do you inspect children's teeth?	3.1%	29.2%	32.3%	35.6%	
Do you refer children that you diagnose with dental caries, to paediatric dentists?	6.9%	16.9%	28.5%	45.4%	2.3%
Do you instruct parents about the importance of tooth brushing, from eruption of the first tooth?	7.7%	26.2%	26.2%	40%	
Do you recommend parents to brush their children's teeth with fluoridated toothpaste?	23.1%	25.4%	24.6%	24.6%	2.3%
Do you explain to parents that their children’s untreated caries are at high risk to develop infections?	37.7%	30.8%	14.6%	13.1%	3.8%
Do you refer 12-month-old infants to paediatric dentists?	39.2%	40%	10%	7%	3.1%
If a child needs a syrup medication, do you recommend giving a sugar-free medication?	56.2%	25.4%	9.3%	5.4%	3.8%

For each question, the percentage of paediatricians that gave an answer on the frequency of the specified behaviour or had no opinion is given.

### Barriers to implementation of AAP guidelines for ECC prevention

Forty-five percent of the respondents indicated that limited time interfered with their implementation of the guidelines, 58.5% stated the absence of oral and dental training, 45.7% stated the lack of confidence in caries risk assessment and 53.2% stated lacking confidence in recognizing early signs of caries. Sixty percent of the participants reported not being familiar with the Israeli Ministry of Health recommendations for caries risk assessment by paediatricians.

### Reasons associated with the practice of caries risk assessment

A positive correlation was found between the paediatrician’s confidence in performing caries risk assessment and its actual practice (*p* = 0.001). No correlation was found between dental training during the paediatrician’s clinical training and confidence in performing caries risk assessment. Eighty-six percent of the participants in the survey expressed their interest in having a continuing education program in paediatric dentistry.

## Discussion

The present study was a survey among paediatricians regarding their knowledge about diagnosis and treatment of early childhood caries. The survey also estimated the implementation of knowledge. We found that despite most of paediatricians found oral and dental health prevention important, they actually did not succeed to perform these measures to all of their young patients. Barriers for that were few: lack of knowledge, lack of confidence and high work load and time shortage this findings are in accordance to findings that were lately published in a systematic rview by Rangel A et al. (15). Paediatricians show interest in participating in continuing education programs regarding early diagnosis and prevention of early childhood caries.

From 145 paediatricians who were offered participation between 2018 and 2020, the questionnaires of 130 were included in this survey, corresponding to 89.6% response rate. The response rates in comparable surveys conducted in the U.S. were 46% (13) and 62% (10). In those surveys, questionnaires were sent by mail, whereas in the current survey they were distributed during a professional convention.

### Dental and oral training during paediatricians’ clinical training

In our study, slightly more than half reported a lack of oral health training. This is consistent with previous reports.

The participants of the current survey reported not having received training during their paediatrics program, in the oral and dental health of infants. In our study, slightly more than half reported a lack of oral health training. Eighty-five percent reported not having received any oral and dental training during their residency; only 15% had received some training in this field This is consistent with previous reports regarding unsatisfactory oral and dental training of paediatricians during their clinical training (10, 11, 13, 14, 16). These findings contrast with the recommendations of the AAP (5). In a U.S. national survey from 2009, 21% of post-residency fellows in paediatrics reported not having received any oral or dental training (12). Sixty-one percent of the participants of the present study thought that the oral and dental training they received was insufficient.

### Dental and oral knowledge among paediatricians

In the current survey, 62% of the paediatricians responded correctly to three or fewer questions of five, which tested their dental and oral knowledge. Only 27% replied correctly to all five questions. These findings suggest a moderate level of knowledge, and lower than expected from a professional who advises parents of infants. Similarly, insufficient knowledge was found in a few surveys from the U.S. The conclusion of those surveys was that oral and dental training should be added to the paediatrics curriculum (10, 13, 16).

Less than half the participants answered correctly regarding fluoride supplements. This was somewhat surprising as according to the Israeli Ministry of Health, fluoride supplements should be recommended for high-risk patients, by a paediatric dentist or paediatrician. However, as guidelines for fluoride supplements have changed over time, a gap in knowledge may be expected, in the absence of routine training on this matter or of self-learning.

### Dental knowledge and the correlation to dental training

Surprisingly, no correlation was found between receipt of dental training during professional training and knowledge scores. The mean score on the five knowledge questions was higher among respondents who received dental training as part of continuing education than among those who did not: 4.10 ± 0.56 vs. 3.36 ± 1.9. From this finding we conclude that during residency programs, paediatricians are busy learning the core of their profession and dental training is only one of the many fields included.

Dental training regarding caries risk assessment and prevention during continuing education, seems much more effective than dental training during basic medical education. It seems that at this later stage the paediatrician is more confident in the main skills of paediatrics and may be more open to acquiring skills and knowledge that are not part of the core of the profession. The higher scores in dental knowledge among respondents who received dental training in continuing education compared to those who did not receive such training suggests that medical schools and paediatric education programs do not provide the dental skills and knowledge that are anticipated from paediatricians. From this finding we understand that dental education must be strengthened during medical school, in post-graduate programs and in continuing education programs.

### Paediatricians’ attitudes regarding promoting dental health among their young patients

Eighty percent of the participants in this survey believed that the paediatrician has a major role in promoting oral and dental health among their young patients. Eighty-seven percent thought that dental inspection should be part of the paediatric physical examination. Eighty-nine percent thought that paediatricians should provide dental caries preventive instruction, as part of routine growth and development follow up. This is in accordance with other surveys (10, 13, 17).

Only 50.8% of the participants in this survey agreed with the AAP guidelines that children should be referred to paediatric dentists by their first birthday. In a survey in the U.S., paediatricians explained that they referred at an older age, as infants might not be able to cooperate in a dental examination (13). Socioeconomic status of the patients affected the time of referral. As dental service for children is free of charge in Israel since 2010, socioeconomic status presumably does not pose an obstacle for early dental referral.

In the current survey, 58.5% of the participants thought that caries risk assessment should be performed by the paediatrician from age 6 months. This finding contrasts with the AAP guidelines that promote prevention. We examined a correlation of this finding with the level of confidence paediatricians have in performing caries risk assessment. As the level of confidence increased, paediatricians’ attitude to performing caries risk assessment was more favourable (*p* = 0.015). Surprisingly, the level of confidence was not correlated to the scope of their dental training (*p* = 0.131). Strengthening the confidence of paediatricians in performing caries risk assessment should be emphasized throughout dental training, in all the professional levels of training (undergraduate, residency and continuing education).

A positive correlation was found between the opinion that caries risk assessment is necessary for every 6-month-old child, and the opinion that all children should be referred to an evaluation by a paediatric dentist by their first birthday (*p* = 0.015). Enhancement of dental awareness and dental knowledge could raise the confidence of paediatricians to perform caries risk assessment and to increased referral of infants to paediatric dentists.

In contrast to our expectation, no significant correlation was found between the paediatricians’ work environment (hospital, private clinic, public clinic, baby clinic) and their attitude toward promoting dental health. The main parameter that seems to influence the attitudes of paediatricians is the degree to which they are updated with the professional literature. In every work environment, some paediatricians are updated. Paediatricians who treat life-threatening situations in hospitals were not less updated than other paediatricians. Efforts should be made to improve knowledge and awareness in every realm of paediatrics.

### Actual clinical performance

Forty-eight percent of the survey’s participants stated that they do not instruct their patients’ parents in preventing dental caries. Among those who do instruct parents, 85% gave oral instructions (and not a written leaflet for example). Among paediatricians in the U.S., 80% reported instructing their patients in dental caries prevention (10, 16). We assume that this difference might be explained by the high workload of paediatricians in Israel, and relates to knowledge and awareness, areas that can be influenced.

Seventy-five percent of those who instructed their patients gave dietary instructions, mainly avoidance of sugar consumption, but only 47% sought information regarding what children drink through bottle feeding. This finding is disturbing, as consumption of beverages that are not water through a bottle is a known risk factor for early childhood caries (3, 4).

Thirty-four percent of those who instructed their patients’ parents, gave oral hygiene instructions. This low proportion is also disturbing, as teeth brushing to infants is not a trivial concept for parents and has a major influence on dental caries risk.

### Dental examinations as part of physical examinations

Only 36% of the participants in the survey reported that they examine their infants’ teeth routinely. This is in contrary to the 87% who agreed that dental examinations are part of paediatric physical examinations. Performing dental examinations as part of physical examinations is highly important, for the promotion of caries risk assessment tailored to every patient.

### Referral to paediatric dentists

Sixty-six (51%) of the respondents reported that they refer patients to paediatric dentists if they diagnose caries. Only 23 (18%) of the responders reported that they refer patients on a routine basis at age 12 months. This finding is coherent with the literature. In a national survey in the U.S., only 14.6% found it necessary to refer patients at age 12 months to paediatric dentists for first examinations (10).

### Explanations to parents on bacterial transmission

Thirty-eight percent of the respondents reported that they do not explain to parents that dental caries pathogens can be transmitted from a major caregiver to an infant’s mouth, while 31% sometimes explain this. This finding contrasts with the finding that 71.5% of the participants in the survey responded correctly regarding the relation between high caries activity in a parent’s mouth and transmission of the disease to a child. Understanding the concept of pathogen transmission from parent to child is crucial for young parents. As most paediatricians understand the aetiology of caries, it is surprising that only a small proportion find it necessary to communicate this important knowledge to parents. The reason may be shortage of time and a high load of patients.

### Clinical performance and the work environment

Though paediatricians from all work environments (hospital, private clinic, public clinic, baby clinic) showed positive attitudes toward promoting oral and dental health, higher proportions of paediatricians who worked in private clinics and baby clinics, compared to those who worked in a hospital environment, reported promoting oral and dental health. This finding was significant (*p* = 0.005 regarding private clinics and *p* = 0.002 for baby clinics); several explanations are possible. Firstly, paediatricians who work in hospital environments deal with life-threatening situations and have a very high workload, while paediatricians who work in private clinics and baby clinics deal with more elective situations and may have more time to perform caries risk assessment and to provide instructions for prevention. Another reason is that paediatricians in baby clinics are especially well trained in treating very young patients and their health needs. This may explain the tendency of these paediatricians to dedicate more time and efforts to dental caries prevention and promoting oral and dental health.

### Barriers to complying with the AAP recommendations

The main difficulties cited in implementing AAP guidelines regarding promoting oral and dental health were lack of awareness to the AAP guidelines regarding oral and dental health (60%), time shortage (46%), and lack of confidence in diagnosing early signs of dental caries (53%) and in risk assessment of dental caries (45%) (13).

Fifteen percent of the respondents reported not referring patients to paediatric dentists due to socioeconomic reasons. This finding is low compared to findings from the U.S. (77.4% and 55.1%) (10, 14). While in the U.S., dentistry is private or covered by insurance; in Israel, since 2010, paediatric dentistry is free of charge for children under age 18 years.

We found that paediatricians with more confidence in risk assessment of dental caries are more likely to perform activities that promote oral and dental health among infants. Pierce et al. found that following 2 h of training, paediatricians were able to diagnose early signs of dental caries with sufficient accuracy to refer to a paediatric dentist (17). Competence in dental caries diagnosis and caries risk assessment can be acquired and improved. Including these topics in continuing education programs for paediatricians is highly important.

### Study limitations

The sample in our study is a convenience sample and is not a representative sample of all the 3,000 licensed paediatricians in Israel.

As in every questionnaire, responder bias should be considered. Paediatricians who cooperated with filling the questionnaire may have been more updated with the guidelines.

Another limitation of the study is that the long length of the questionnaire, which included 42 questions, may have discouraged some paediatricians from participating. Nonetheless, the response rate of 90% was high.

All potentials work places for paediatricians were represented in the current survey: hospitals, private practice, the national health services and baby clinics. Most (64.7%) of the respondents’ patients were younger than 36 months. More than half the respondents worked in hospitals. We assume that in hospitals, most patients have severe diseases or life-threatening conditions, and that caries risk assessment is not an urgent matter.

### Conclusion

1. In the present study more than half reported a lack of oral health training.
2. Dental and oral knowledge among paediatricians is moderate and lower than expected from a professional who advises parents of infants.
3. Most paediatricians agreed that dental inspection and providing dental caries preventive instruction should be part of the paediatric physical examination.
4. Oral health and dental knowledge should be incorporated into the paediatric medicine curriculum, and especially in continuing education programs for paediatricians.
5. Many paediatricians do not perform dental caries risk assessment and counselling, due in part to their high workload and lack of knowledge.

## Data Availability

The original contributions presented in the study are included in the article/**Supplementary Material**, further inquiries can be directed to the corresponding author.
