# Supplementary material for: Promoting oral and dental health in early childhood - knowledge, views and current practices among paediatricians in Israel
Source: Front Pediatr. 2023 Jan 6;10:956365. doi: 10.3389/fped.2022.956365 (PMC9853542; doi:10.3389/fped.2022.956365)
Supplement: Supplementary file 1 [file Datasheet1.docx]

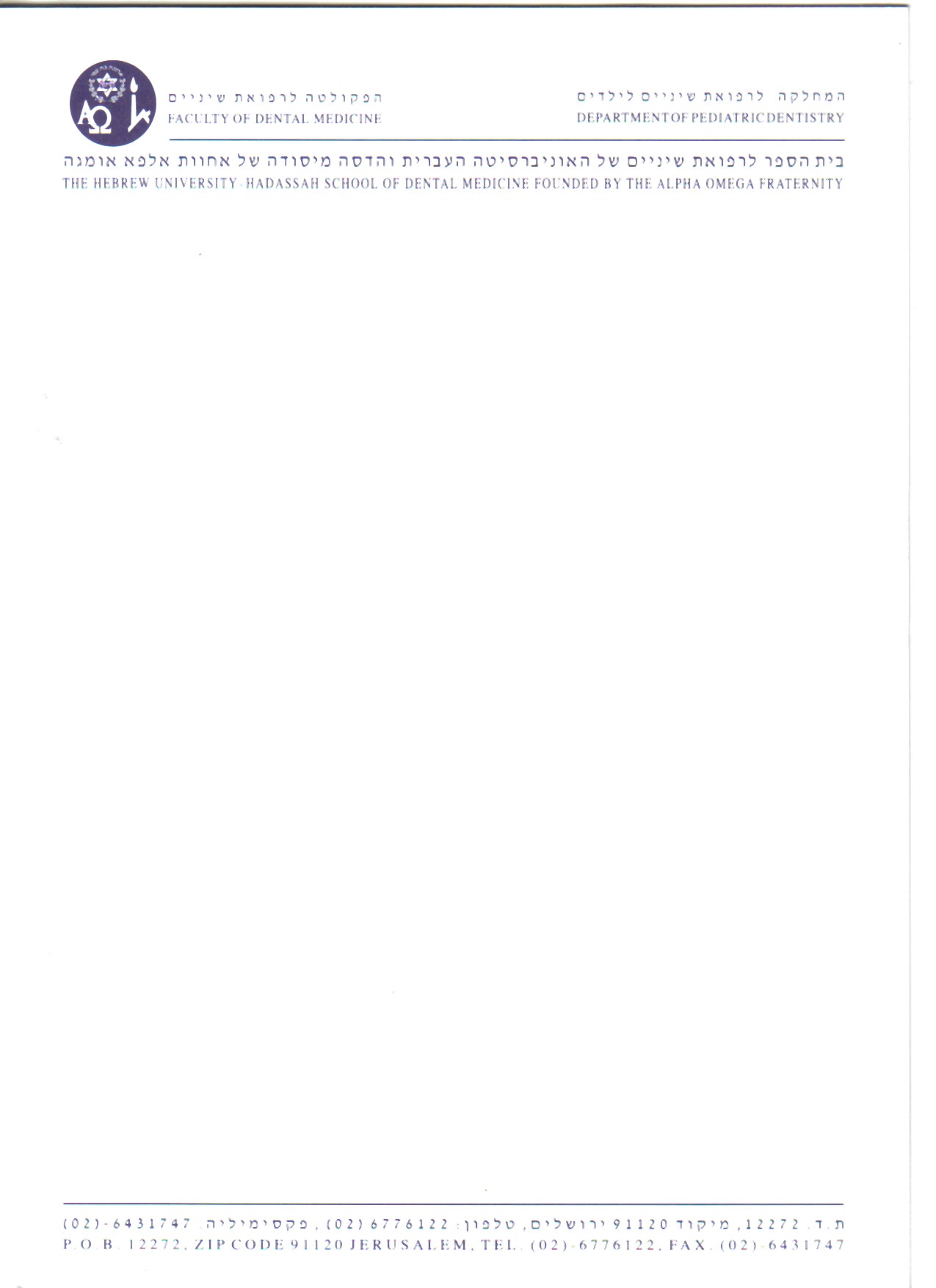


**Part A**

Answer the following questions as they pertain to you, either by marking ‘X’ or filling the gray square.

1. Age:

2. Gender:  Female  Male

3. Year of residency graduation:

4. Place of residency graduation (if Israel, please state the name of the university from which

you graduated. If abroad, please state the name of the country):

5. Number of years practicing:

6. Current workplace (please check all that apply):

Private practice

Independent practice

Health Maintenance Organization (Kopat Hulim)

Family Health Center (Tipat Halav)

Hospital

Other (please elaborate)

7. Which city do you work in?

8. How many patients do you see on an average day?

Among those, what is the percentage of 3 years old and younger?

**Part B**

During your studies, did you receive any training in the field of dentistry? Please mark ‘X’ for the relevant answers.

|  | **Training Type** | **I did not receive training** | **One course** | **Several courses** |
| --- | --- | --- | --- | --- |
| 9. | Medical school |  |  |  |
| 10. | Pediatric residency |  |  |  |
| 11. | Continuing education |  |  |  |

12. Are you familiar with the AAP (American Academy of Pediatrics) oral and dental health guidelines for children and toddlers?  Yes  No

Please indicate your level of agreement with the following statements, by marking ‘X’ in the correct place.

|  | **Statement** | **Agree** | **Disagree** |
| --- | --- | --- | --- |
| 13. | Toddlers who are bottle-fed are the only group at risk of developing early childhood caries (ECC) |  |  |
| 14. | In areas with partial water fluorination, it is custom to give toddlers fluoride additives |  |  |
| 15. | It is known that caries causing bacteria can transfer from mother to baby |  |  |
| 16. | The AAP recommendation is for toddlers to have their first pediatric dentist visit at one year old |  |  |
| 17. | Preterm babies are at higher risk to develop caries |  |  |

Please indicate your level of agreement with the following statements pertaining to well-child visits done by pediatricians, by marking ‘X’ in the correct place. Please mark only one answer.

|  | **Statement** | **Disagree** | **Partially disagree** | **Agree** |
| --- | --- | --- | --- | --- |
| 18. | Teeth examination is an integral part of the overall physical exam |  |  |  |
| 19. | All patients six months and older should be routinely assessed for early childhood caries (ECC) |  |  |  |
| 20. | Caries prevention guidance should be routinely provided to parents during well-child visits |  |  |  |
| 21. | Pediatricians have a central role in promoting oral and dental health within their patient population |  |  |  |
| 22. | Any toddler reaching the age of one year old should be referred to a pediatric dentist for routine follow up |  |  |  |

23. Do you provide routine guidance for parents regarding their child/children’s oral and dental health maintenance?

Yes  No  Sometimes

24. If you marked ‘Yes’ in the previous question, in what manner do you provide the guidance?

You may mark more than one correct answer.

In writing  Oral explanation  Demonstration

The following statements pertain to the routine follow-up you provide for toddlers until the age of 3. Please indicate to what extent these statements are true for you on a scale of 1 (=never) to 4 (=always). Please mark only one answer.

|  | **Statement** | **Never** | **Sometimes** | **Mostly** | **Always** |
| --- | --- | --- | --- | --- | --- |
| 25. | I inquire information as to the toddler’s habit of bedtime bottle, that isn’t water |  |  |  |  |
| 26. | I guide the parents to avoid giving the toddler sweetened foods and drinks |  |  |  |  |
| 27. | I examine the toddler’s teeth |  |  |  |  |
| 28. | If I detect caries during the toddler examination, I refer the parents to a pediatric dentist's consultation |  |  |  |  |
| 29. | I guide the parents on the importance and technique of brushing the toddler’s teeth, starting from the eruption of the first tooth. |  |  |  |  |
| 30. | I guide the parents to brush the toddler’s teeth with a fluoride-containing toothpaste |  |  |  |  |
| 31. | I explain that if the parents have untreated caries, there is an increased chance that their toddler will suffer from the same issue |  |  |  |  |
| 32. | I refer toddlers who reached the age of one year to a pediatric dentist follow-up |  |  |  |  |
| 33. | If a toddler is prescribed medication in the form of a syrup, I provide information about syrups that are sweetened with sugar substitutes |  |  |  |  |
| 34. | If a toddler fell and injured his teeth/mouth area, I will refer him/her to a pediatric dentist even if he doesn’t complain of pain |  |  |  |  |

As part of the routine follow-up you provide for toddlers until the age of 3, which of the following will cause interference with teeth examination, providing the parents with guidance regarding caries prevention as well as referral to pediatric dentist follow-up when the toddler reaches one year of age?

35. Due to time limitations, I’m forced to skip caries prevention guidance to the parents

Yes  No

36. I have a moral issue with referring patients from low socioeconomic status to a dentist.

Yes  No

37. I do not feel that during my medical school studies or residency I have been provided with

adequate training to detect the onset of caries and ways to prevent them.

Yes  No

38. Other reasons, please elaborate

39. To which extent are you confident in your ability to assess the risk factors for caries?

Not at all confident

Somewhat confident

Fairly confident

Fully confident

40. To which extent are you confident in your ability to assess the onset of caries?

Not at all confident

Somewhat confident

Fairly confident

Fully confident

41. Are you familiar with Israeli guidelines in the field of oral and dental health?
  Yes  No

42. If you were offered a continuing education program in the field of preventative dentistry for

preschoolers, would you be interested?
  Yes  No
