# Supplementary material for: Promoting oral and dental health in early childhood - knowledge, views and current practices among paediatricians in Israel
Source: Front Pediatr. 2023 Jan 6;10:956365. doi: 10.3389/fped.2022.956365 (PMC9853542; doi:10.3389/fped.2022.956365)
Supplement: Supplementary file 2 [file Datasheet2.docx]

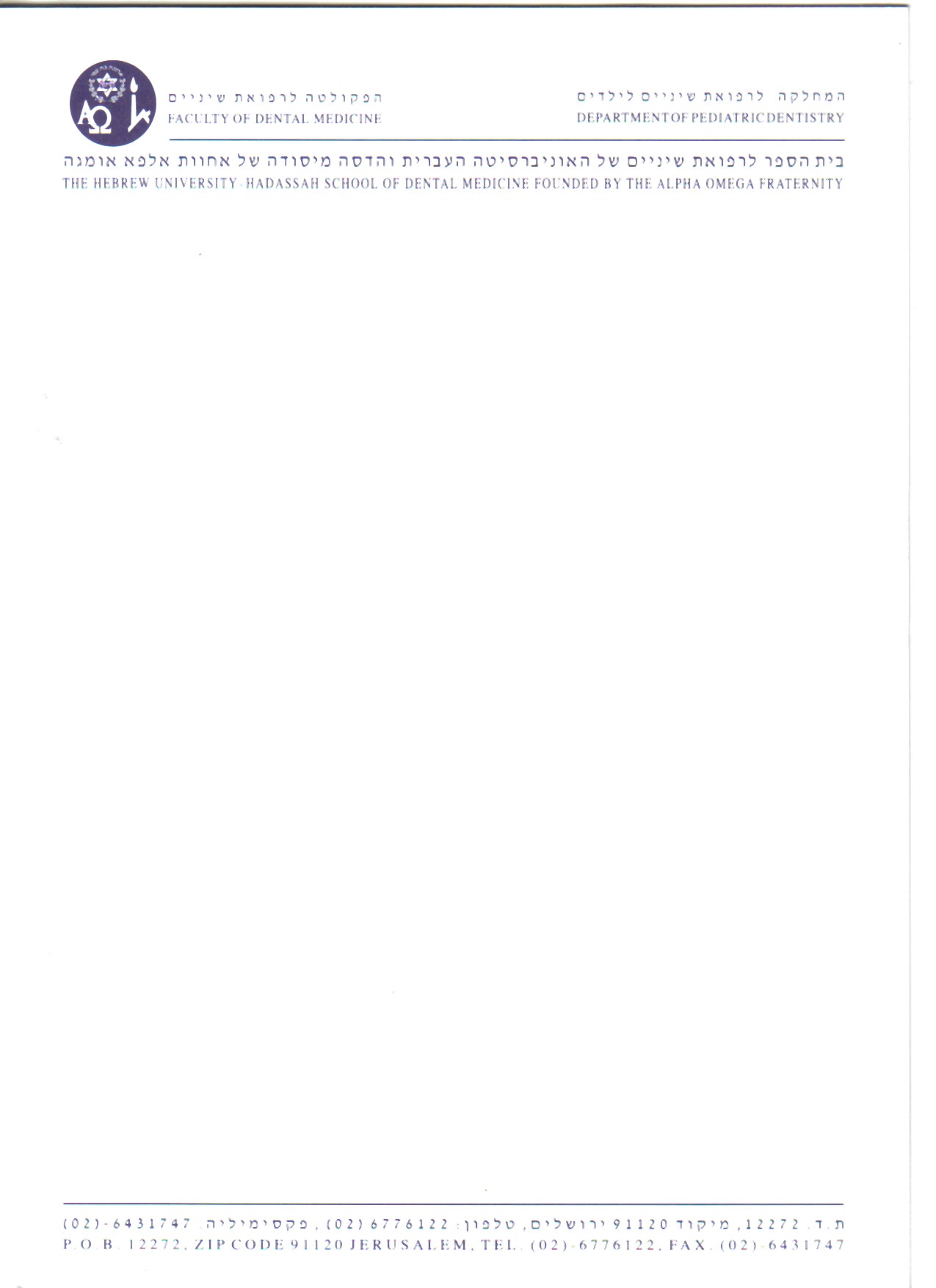


**חלק א'**

לפניך מס' שאלות. בחלקן יש לסמן ב-'X' את התשובה המתאימה או למלא תשובתך במלבן האפור

1. בן/ת כמה את/ה?
2. מין:  1. זכר  2. נקבה
3. באיזו שנה סיימת את ההתמחות:
4. היכן סיימת את ההתמחות (אם בארץ –באיזו אוניברסיטה, אם בחו"ל – יש לציין באיזו מדינה):
5. כמה שנים אתה עובד/ת במקצוע ?
6. מה מקום העבודה הנוכחי (ניתן לסמן יותר מתשובה אחת) :

1. מרפאה פרטית.

2. מרפאה עצמאית.

3. קופ"ח

4. טיפת חלב

5. בי"ח

6. אחר, פרט/י

1. באיזו עיר אתה עובד/ת?
2. כמה מטופלים אתה בודק/ת ביום בממוצע?      . מתוכם מה אחוז המטופלים עד גיל 3?


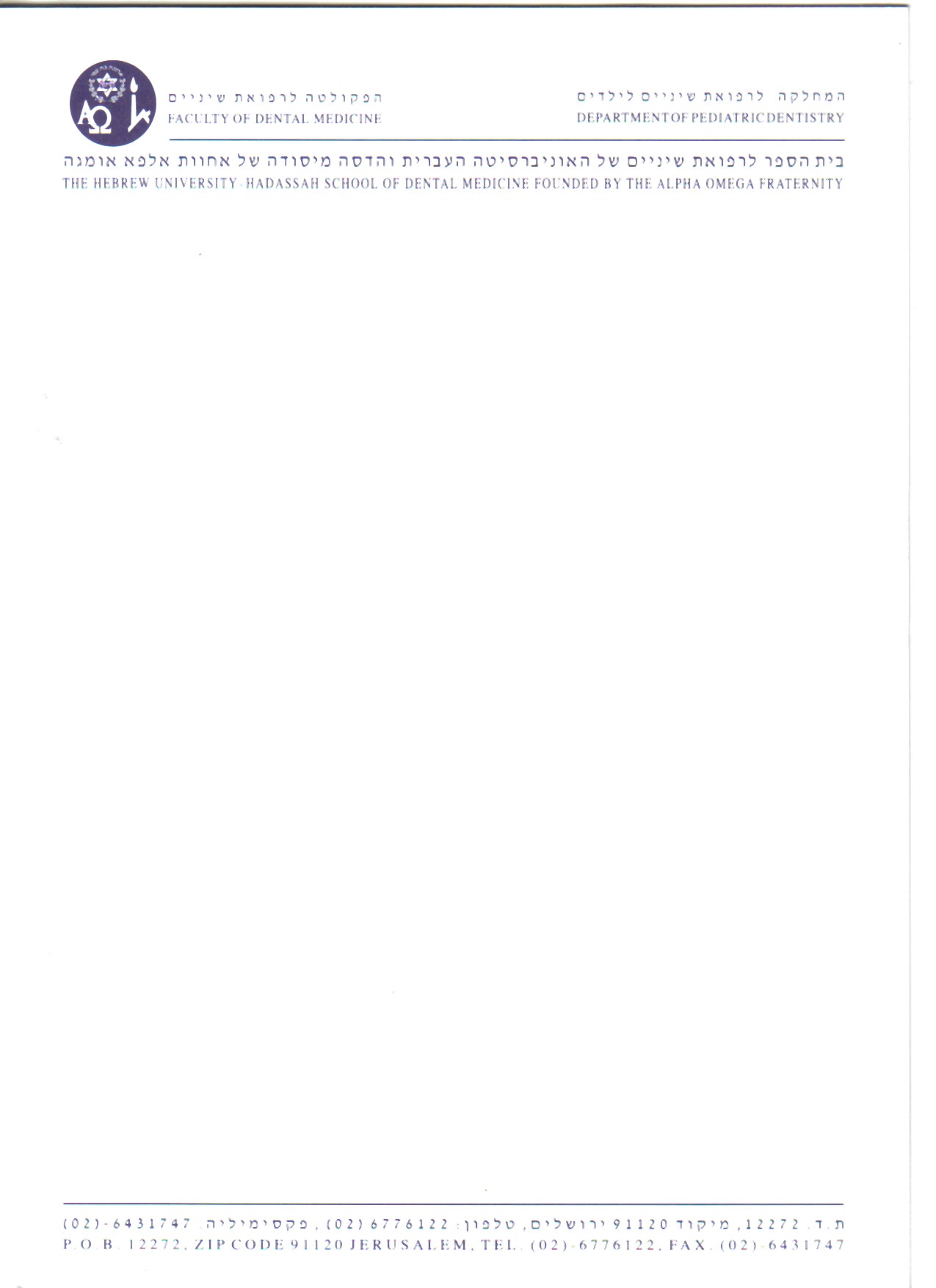

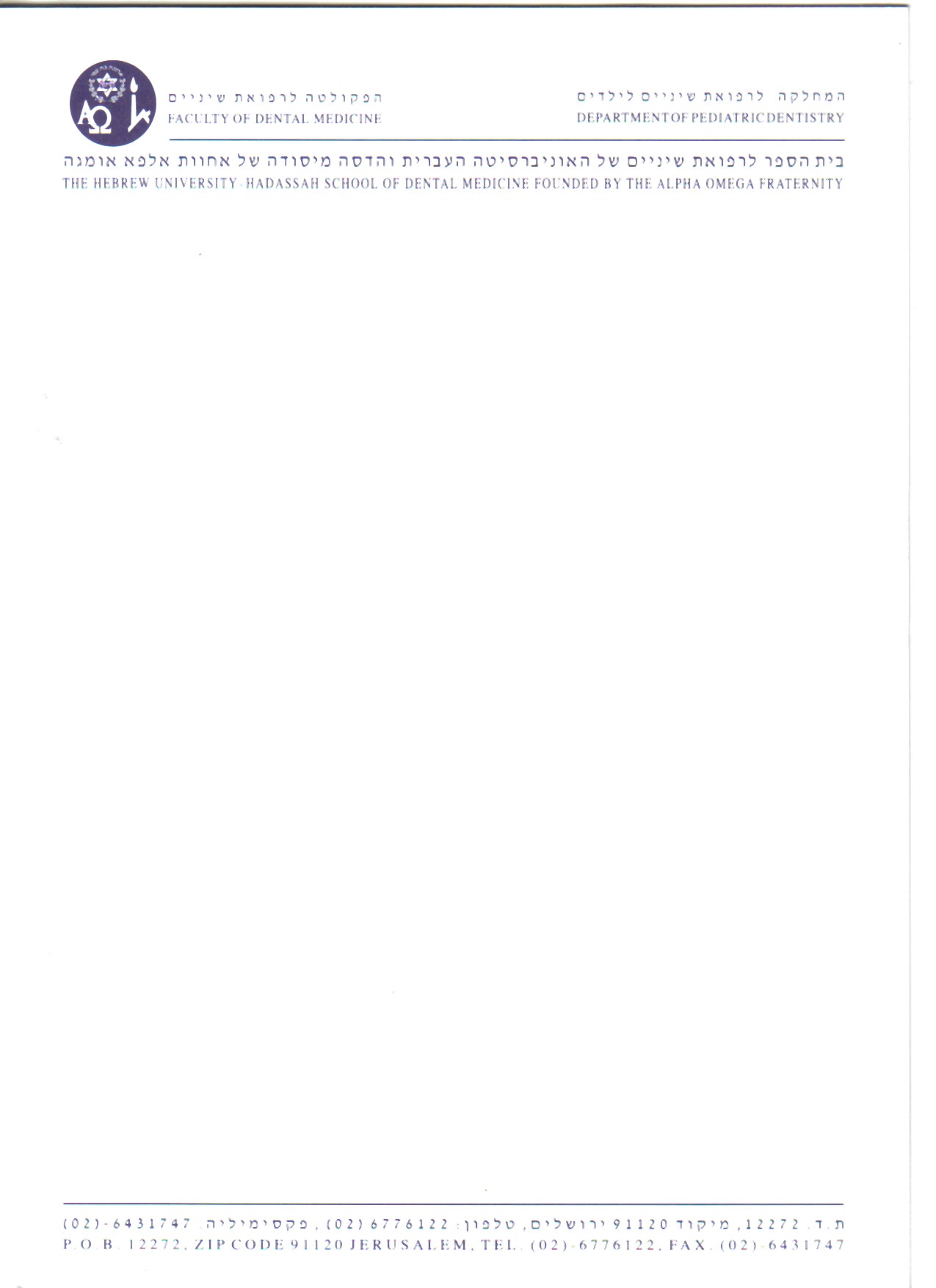
**חלק ב'**

האם עברת במסגרת לימודיך הכשרה כלשהי בתחום רפואת שיניים? יש לציין ב-'X' את משך ההכשרה בכל מסגרת לימודית שעברת.

| **3**  **מס' קורסים** | **2**  **קורס אחד** | **1**  **לא הוכשרתי** | **שאלה** | **מס' פריט** |
| --- | --- | --- | --- | --- |
|  |  |  | לימודי רפואה |  |
|  |  |  | התמחות ברפואת ילדים |  |
|  |  |  | השתלמות /לימודי המשך |  |

12. האם את/ה מכיר/ה את הנחיות ה-AAP (American Academy of Pediatrics) בתחום בריאות הפה והשיניים של ילדים ופעוטות?

1. כן

2. לא

לפניך מספר הצהרות. ציינ/י לגבי כל אחת האם את/ה מסכימ/ה או לא. יש לסמן 'X' במקום המתאים.

| **2**  **לא מסכים** | **1**  **מסכים** | **שאלה** | **מס' פריט** |
| --- | --- | --- | --- |
|  |  | רק פעוטות הניזונים מבקבוק מפתחים עששת הגיל הרך (ECC) . |  |
|  |  | באזור עם הפלרה חלקית, מקובל לתת לפעוטות תוספי פלואוריד. |  |
|  |  | ידוע שחיידקים הגורמים לעששת יכולים לעבור מהאם לתינוק. |  |
|  |  | ה-AAP ממליץ להפנות פעוטות לבדיקה ראשונה אצל רופא שיניים לילדים בגיל שנה. |  |
|  |  | תינוקות אשר נולדו בטרם עת (Preterm) נמצאים בסיכון גבוה לפתח עששת . |  |


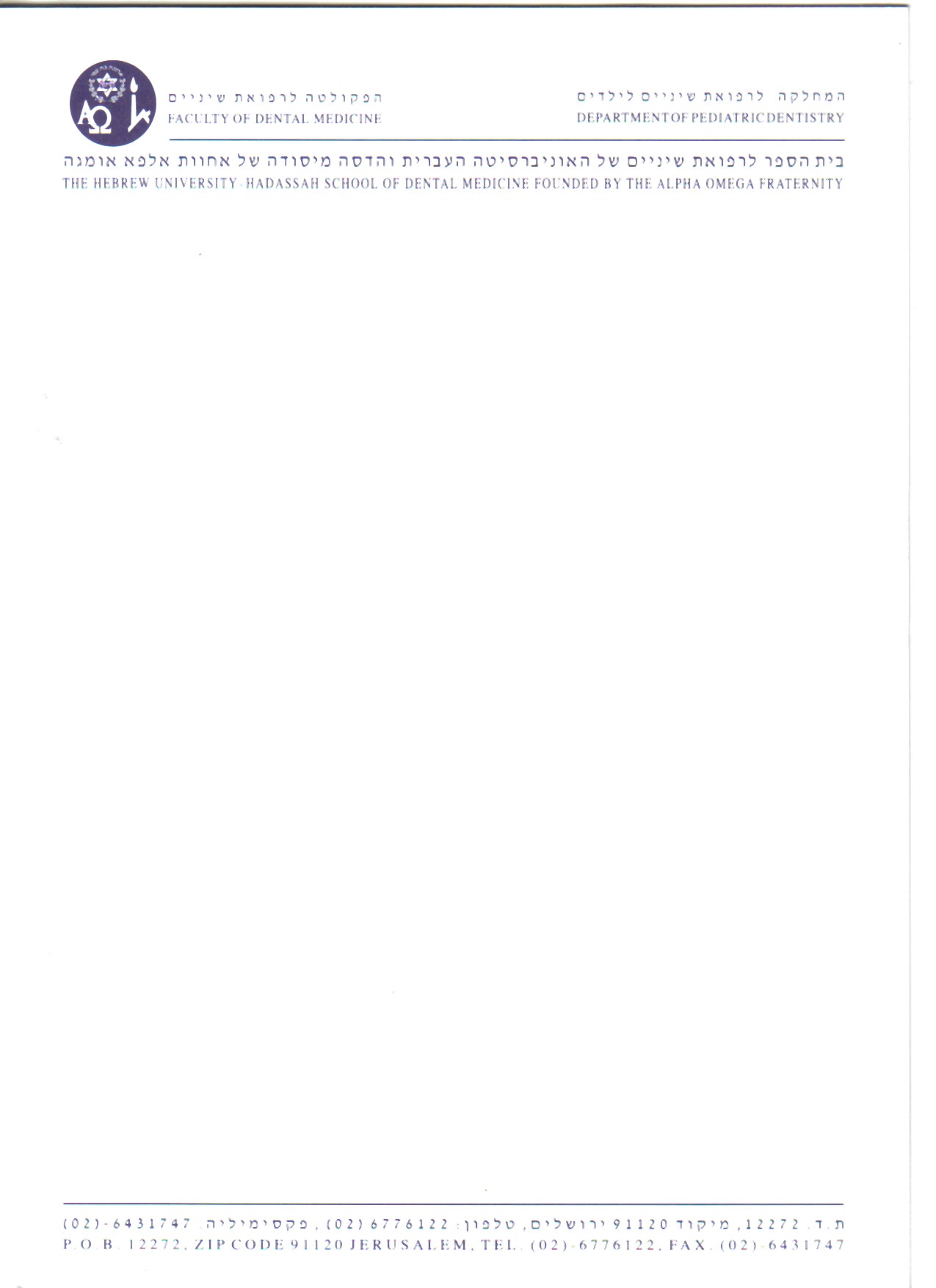

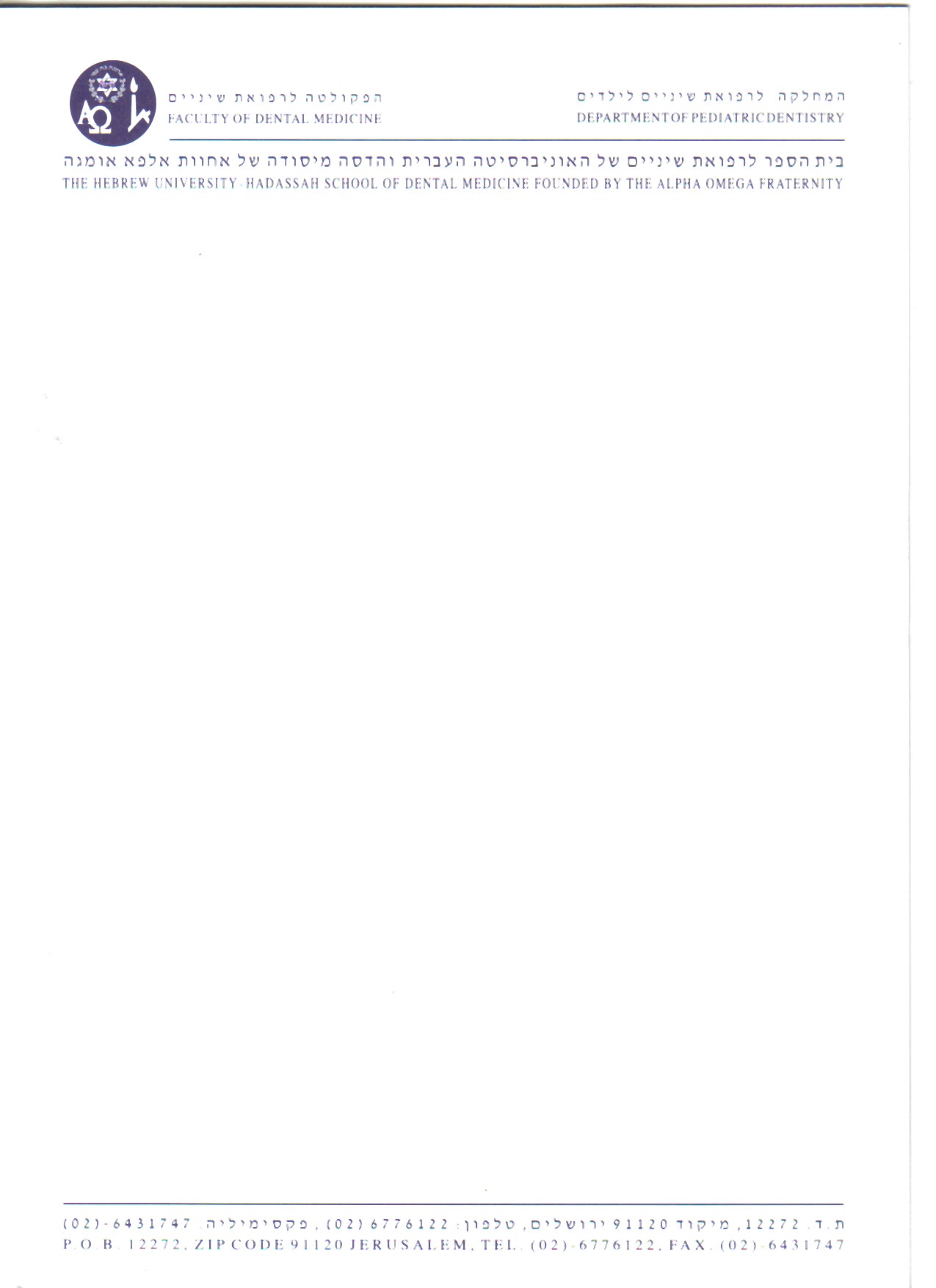


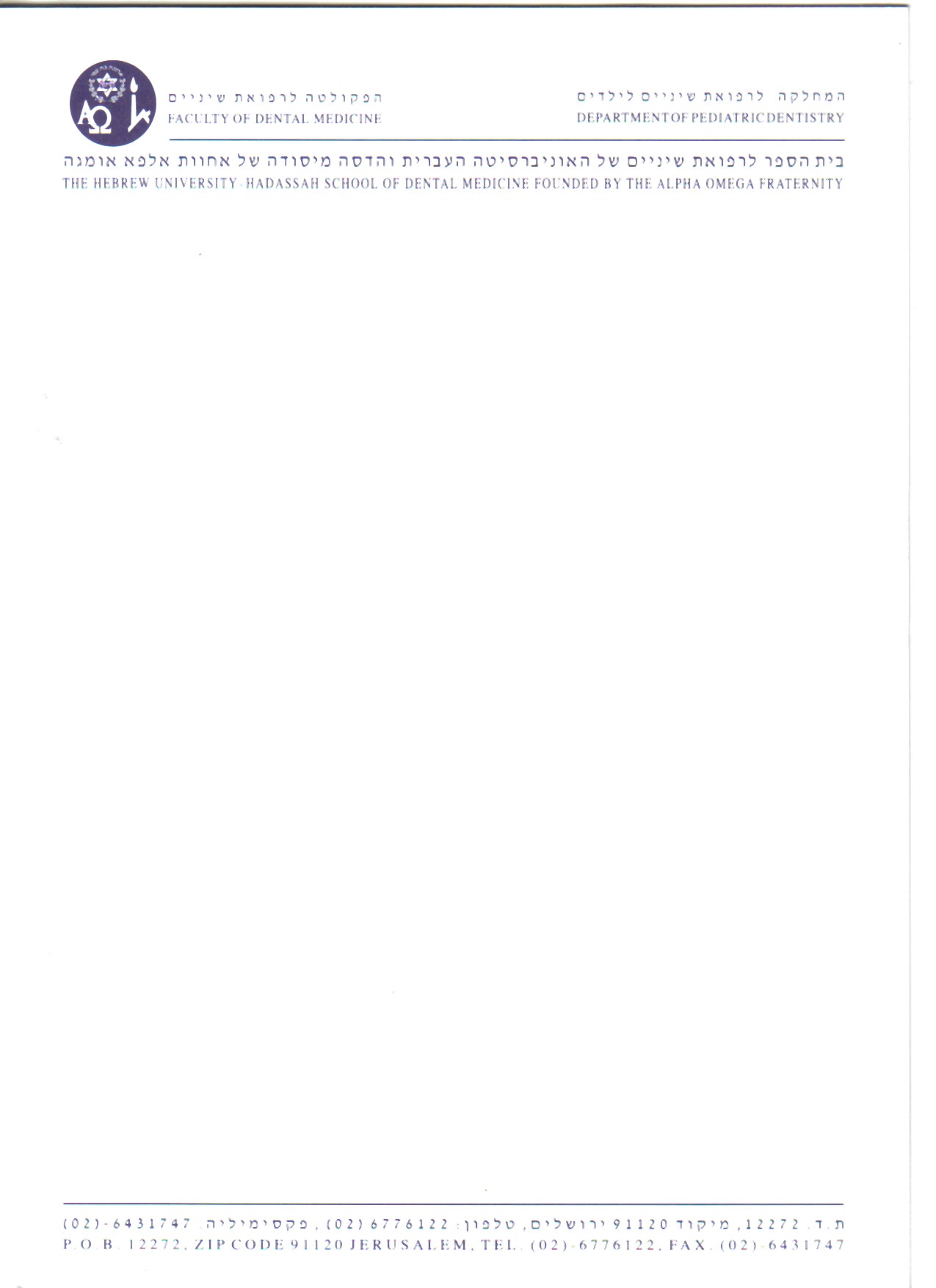

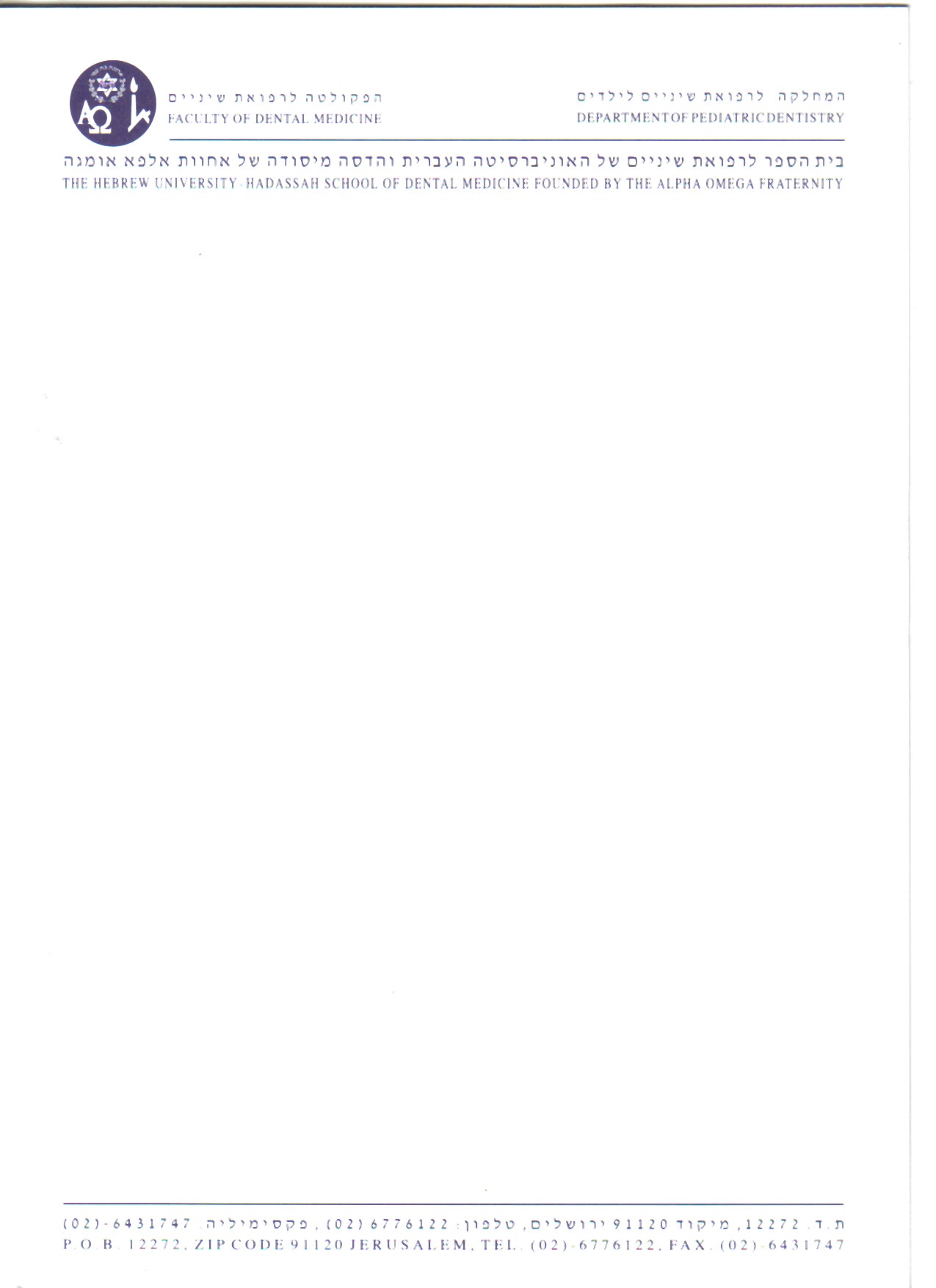
לפניך מס' הצהרות המתייחסות למעקב התקופתי (well child visits) שרופאי ילדים עורכים לפעוטות. עליך לציין באיזו מידה אתה מסכימ/ה עם כל אחת מהן. התשובות העומדות לרשותך הן: מסכים / לא כל-כך מסכים / לא מסכים. יש לסמן 'X' במקום המתאים, תשובה אחת בלבד לכל הצהרה.

| **3**  **מסכים** | **2**  **לא כל-כך מסכים** | **1**  **לא מסכים** | **שאלה** | **מס' פריט** |
| --- | --- | --- | --- | --- |
|  |  |  | הסתכלות על השיניים הינה חלק אינטגרלי מהבדיקה הפיזיקלית. |  |
|  |  |  | יש לבצע הערכת סיכון לעששת הגיל הרך (ECC) באופן שגרתי לכל המטופלים החל מגיל 6 חודשים. |  |
|  |  |  | יש לתת הדרכה למניעת עששת באופן שגרתי במהלך המעקב התקופתי. |  |
|  |  |  | לרופאי ילדים תפקיד מרכזי בקידום בריאות השיניים של מטופליהם. |  |
|  |  |  | יש להפנות כל פעוט לרופא שיניים לילדים בהגיעו לגיל שנה. |  |

1. האם את/ה נוהג/ת להדריך את ההורים כיצד לשמור על בריאות השיניים של ילדיהם?

1. כן

2. לא

3. לפעמים

1. באם כן, כיצד את/ה מדריכ/ה את ההורים? (יש לסמן 'X' במקום המתאים. ניתן לסמן יותר מתשובה אחת)

1. ע"י הסבר בכתב.

2. ע"י הסבר בע"פ.

3. ע"י הדגמה.

השאלות הבאות מתייחסות למעקב התקופתי שאת/ה עורכ/ת לפעוטות עד גיל 3. יש לציין באיזו מידה מ-1 עד 4, את/ה נוהג/ת לבצע את הפעולות הבאות? (1=אף פעם, 4=תמיד)

| **4**  **תמיד** | **3**  **לרוב** | **2**  **לפעמים** | **1**  **אף פעם** | **שאלה** | **מס' פריט** |
| --- | --- | --- | --- | --- | --- |
|  |  |  |  | האם את/ה מברר האם הילד נרדם בד"כ עם בקבוק המכיל כל משקה אחר מלבד מים? |  |
|  |  |  |  | האם את/ה מדריכ/ה את ההורים להימנע מלתת לילד משקאות ומאכלים ממותקים? |  |
|  |  |  |  | האם את/ה בודק/ת את שיני הילד? |  |
|  |  |  |  | האם את/ה מפנה מטופל שאבחנת כסובל מעששת לרופא שיניים לילדים? |  |
|  |  |  |  | האם את/ה מדריכ/ה את ההורים לגבי האופן והחשיבות של צחצוח שיני הילד כבר מבקיעת השן הראשונה? |  |
|  |  |  |  | האם את/ה ממליצ/ה להורים לצחצח את שיני הילד במשחת שיניים לילדים המכילה פלואוריד? |  |
|  |  |  |  | האם את/ה מסביר/ה להורים כי במידה ויש להם מוקדי עששת לא מטופלת יש סיכוי כי גם לילדיהם יהיו? |  |
|  |  |  |  | האם את/ה מפנה פעוט בהגיעו לגיל 12 חודשים לבדיקה אצל רופא שיניים לילדים? |  |

| **4**  **תמיד** | **3**  **לרוב** | **2**  **לפעמים** | **1**  **אף פעם** | **שאלה** | **מס' פריט** |
| --- | --- | --- | --- | --- | --- |
|  |  |  |  | כשילד זקוק לתרופה הניתנת כסירופ, האם אתה ממליץ להורים לבחור בסירופ שמומתק בתחליף סוכר? |  |
|  |  |  |  | מפנה לרופא שיניים ילד שנפל ונחבל בשיניו, גם אם אינו מתלונן על כאבים? |  |

במסגרת המעקב התקופתי שאת/ה עורכ/ת לפעוטות עד גיל 3, מה מהבאים מפריע לך לערוך בדיקת שיניים, להדריך את ההורים

למניעת עששת ולהפנות פעוט לרופא שיניים לילדים בהגיעו לגיל שנה? (יש לסמן 'X' במקום המתאים).

35. בשל מגבלת הזמן אני נאלצ/ת לוותר על הנחיות להורים למניעת עששת בפעוט -

1. כן

2. לא


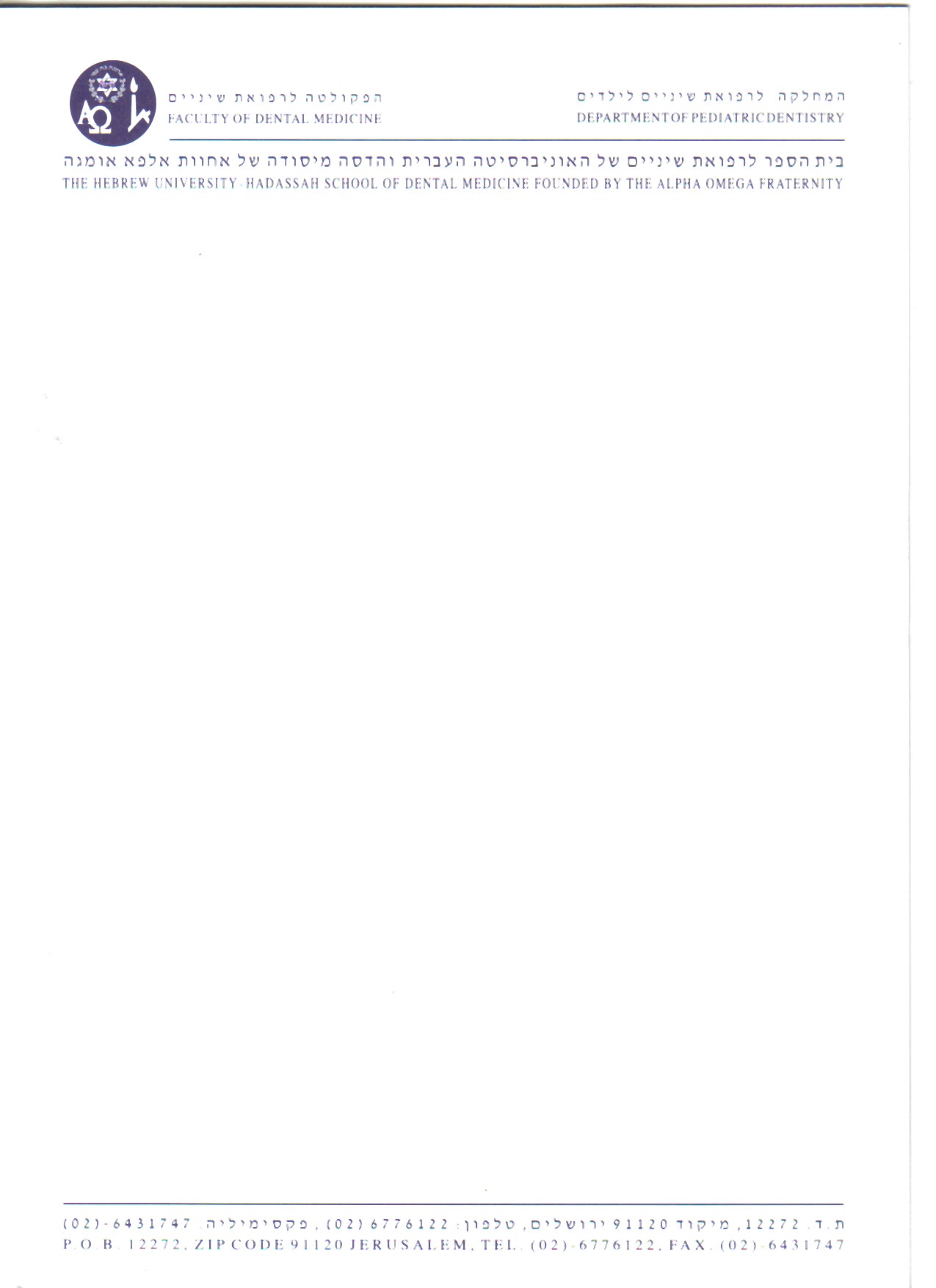
. 36יש לי קושי מוסרי להפנות מטופלים ממעמד סוציואקונומי נמוך לרופא שיניים –

1. כן

2. לא

37. אינני מרגיש/ה שבמסגרת לימודי הרפואה וההתמחות עברתי את ההכשרה הנדרשת לשם איבחון עששת התחלתית והדרכה למניעתה -

1. כן

2. לא

1. סיבה אחרת, פרט/י
2. באיזו מידה את/ה חש/ה ביטחון לבצע הערכת סיכון לעששת?

1. כלל לא.

2. במידה מועטה.

3. במידה בינונית.

4. במידה רבה

1. באיזו מידה את/ה חש/ה בטחון ביכולתך לאבחן עששת בשלביה הראשונים?

1. כלל לא.  3. במידה בינונית.

2. במידה מועטה.  4. במידה רבה

1. האם את/ה מכיר/ה הנחיות ישראליות בתחום בריאות השיניים?

1. כן

2. לא

1. אילו היו מציעים לך להשתתף בקורס השתלמות בנושא רפואת שיניים מונעת לגיל הרך, האם היית מצטרפ/ת אליו?

1. כן

2. לא

**תודה על שיתוף הפעולה !**
